# OA28 Case report: chronic myeloid leukemia in childhood

**DOI:** 10.1093/rap/rkae117.028

**Published:** 2024-11-05

**Authors:** Aryan Jalal

**Affiliations:** Kurdistan Higher council for medical specialties, Rheumatology department, Erbil, Iraq

## Abstract

**Introduction:**

Myeloproliferative neoplasms (MPNs) are rare hematological malignancies. The median age at diagnosis of chronic myelogenous leukemia (CML) is 60 to 65 years CML is the commonest MPN characterized by extreme leukocytosis, myelocyte bulge, basophilia, eosinophilia in peripheral blood and presence of underlying Philadelphia chromosome as a cytogenetic abnormality or BCR-ABL transcript as a molecular abnormality. It is mainly a disease of adults, the incidence is rare in children of which it accounts for only 3% of leukemias in this age group, 9% in adolescents, characterized by acquisition of Philadelphia chromosome (Ph) by the leukemic cells in adults and children alike.

**Case description:**

Fifteen-years-old male lives in Kurdistan, his condition started 14^th^ of February 2021 and he came to Rheumatology outpatient clinic region. His main complaint was pain and swelling of the right knee joint for two days with some difficulty of walking, associated with high grade intermittent fever with decreased appetite with no extra articular manifestation. Family history was negative for rheumatological and hematological diseases. On clinical examination (Blood pressure 120/70, PR 90 beat per minute, temperature 37.7, respiratory rate 18), there were two cervical lymphadenopathy of right side anterior group of cervical lymph node size about 1cm mobile not tender movable, bilateral epitrochlear lymph node enlargement, abdominal examination soft non tender, there was huge splenomegaly about 20 cm reaching umbilicus not tender, cardiovascular, respiratory and neurological examinations were unremarkable. On musculoskeletal examination right knee joint line was tender with sweeping sign positive for fluid but patellar tab was negative, with no limitation of movement, other joints normal on examination. The patient was sent for blood investigations including complete blood picture (CBP), which shows leukocytosis 305 (normal reference 3.5-10.0), Hemoglobin 10.2 g/dl (11.5-16.5), platelet 296 (100-400), ESR 35 mm/hr (<20), CRP 8.13 mg/l (0-5), serum Vitamin D 7.34 (sufficient 30-70), Serum uric acid was 2.76 mg/dl (normal range 3.4–7.2 mg/dl), Echocardiography was normal, ultrasound of abdomen shows markedly enlarged spleen 21 cm reaching the pelvis homogenous in texture, no inguinal lymphadenopathy. We suspected blood malignancy (leukemia) and referred the patient to hematology department, after evaluation they investigated bone marrow biopsy, which shows (a tiny fragment of marrow core with cellularity approaching 90%,erythroid series were increase with many large from granulocyte series were increased while megakaryocyte were a adequate). They sent for BCR-ABL which displayed a positive level more than upper Loq.

**Discussion:**

The patient was started on Hydroxyurea 500 mg for five days after availability of BCR-ABL, imatinib was added on day 10, his WBC reduced; hydroxyurea was stopped, patient continue only on imatinib 400 mg. The patient responded to treatment with the tyrosine kinase inhibitor drug, imatinib, with WBC recovery at six weeks later it reduced to 5.2 which was normal.

There are two major types of leukemia. Acute Lymphoblastic Leukemia (ALL) accounts for almost three quarters of diagnosis and acute non-lymphoblastic comprises 19%. CML is very rare in this age group in childhood. Leukemia are a group of neoplasms that arise from the malignant transformation of hematopoietic cells. Leukemia are classified according to the cell types involved (myeloid or lymphoid) and as acute or chronic based upon the natural history of the disease. The majority of paediatric cases are diagnosed over the age of five.

The I-CML-Ped Study reported a higher total WBC count (mean 250 × 109 /l), lower hemoglobin, and higher peripheral circulating blasts than the adults. (10-12). Similar results were also reported by Chandra D et al. The present case is 15-year-old male who presented with joint pain, fever, splenomegaly, TLC of 305 x 10³/µL.

The risk of tumor lysis syndrome is low in a paediatric CML; however, the patients are treated with oral hydration, hydroxyurea and allopurinol until the diagnosis is established. Imatinib is an effective tyrosine kinase inhibitor (TKI) in the paediatric age group which trials reveals up to 89% complete hematological response occur within three months of starting treatment and 97% event-free survival in 18 months. The side effect that is common with imatinib therapy include nausea, diarrhoea, vomiting, bone pain, and cytopenia’s.

**Key learning points:**

• This form of presentation of CML has not been reported in children. The incidence of leukemia among children younger than 15 years of age has increased in the past 20 years, with the trend reflecting an increase in incidence of Acute Lymphoblastic Leukemia (ALL) during this period. We report a case who presented with extreme leukocytosis, which, on further investigations, turned out to be CML (BCR-ABL+, by reverse transcription PCR (RT-PCR). Children with CML are exposed to tyrosine kinase inhibitors during their growth years leading to higher burden of morbidities, which can be different from those of adults, and require careful monitoring. Any joint pain in children should be thoroughly examined in patients, concerns should be explored and the patients should be sent for further investigation in order to exclude malignancy.

